# Supplementary material for: A Michael Acceptor Analogue, SKSI-0412, Down-Regulates Inflammation and Proliferation Factors through Suppressing Signal Transducer and Activator of Transcription 3 Signaling in IL-17A-Induced Human Keratinocyte
Source: Int J Mol Sci. 2021 Aug 16;22(16):8813. doi: 10.3390/ijms22168813 (PMC8396041; doi:10.3390/ijms22168813)
Supplement: Supplementary file 1 [file ijms-22-08813-s001.zip › ijms-1300003-supplementary.pdf]

Supplementary Information

A Michael acceptor analogue, SKSI-0412, down-regulates inflammation and proliferation factors through suppressing signal transducer and activator of transcription 3 signaling in IL-17A-induced human keratinocyte

Aram Kim[1,†], Seungbeom Lee[2,†], Jung U shin[1], Seung Hui Seok[1], Young-Ger Suh[2]*, and Dong Hyun Kim[1]*

1. Department of Dermatology, CHA Bundang Mediacal Center, School of Medicine, CHA University, Seongnam-Si, Gyeonggi-do, Republic of Korea

2. College of Pharmacy, CHA University, Seongnam-Si, Gyeonggi-do, Republic of Korea

*Correspondence: terios92@hanmail.net (D.H.K.); ygsuh@cha.ac.kr (Y.G.S.)

†These first authors contributed equally to this work

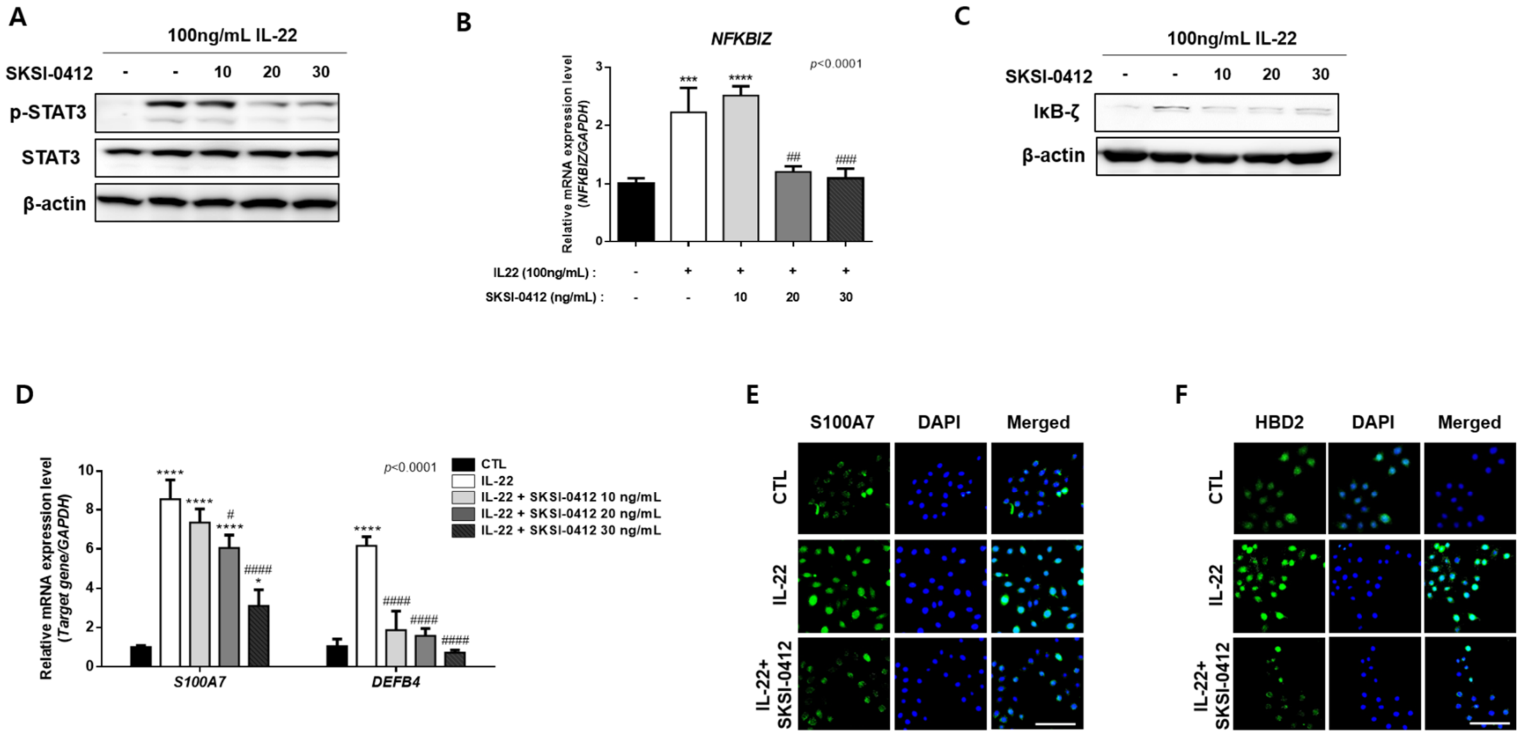

Supplementary Figure S1. SKSI-0412 downregulates IκBζ, S100A7 and HBD-2 by inhibition of STAT3 phosphorylation. (A) SKSI-0412 suppress phosphorylation of STAT3 in IL-22-induced keratinocyte for 30 mins. (B) Gene and (C) protein expression of IκBζ is down-regulated by SKSI-0412 in IL-22-induced keratinocyte for 6 hrs. ***P < 0.001, ****P< 0.0001 compared with non-treated control; ## P<0.01, #### P<0.0001 compared with IL-22 treatment sample, ANOVA one-way test. (D) SKSI-0412 decrease expression of *S100A7 and DEFB4* mRNA in IL-22-induced Keratinocytes. *P < 0.05, ****P< 0.0001 compared with non-treated control; # P<0.05, #### P<0.0001 compared with IL-22 treatment sample, ANOVA one-way test. The protein expression of (E) S100A7 and (F) HBD2 was confirmed by immunofluorescence staining of S100A7(green), HBD2(green) and DAPI(blue). *Scale bar* =100 μm.

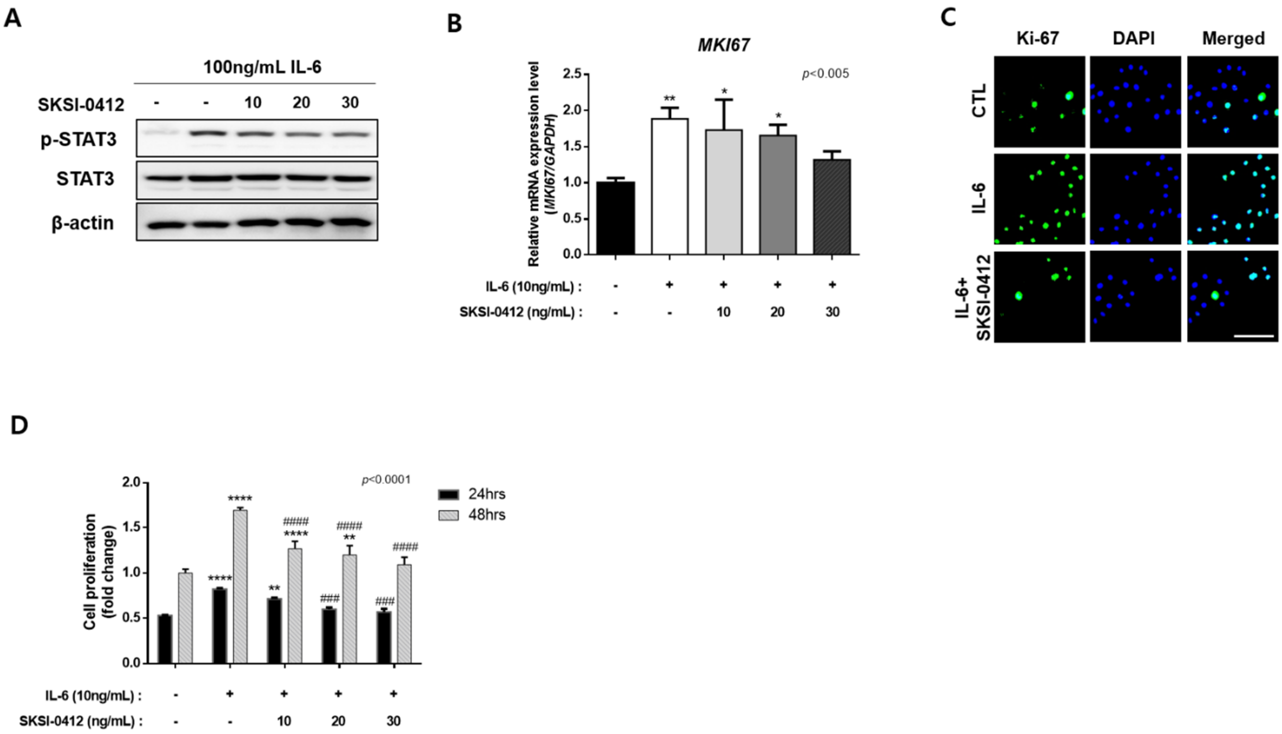

Supplementary Figure S2. SKSI-0412 downregulates cell proliferation by regulation of IL-6/STAT3 signaling pathway. (A) SKSI-0412 suppress phosphorylation of STAT3 in IL-6-induced keratinocyte for 5 mins. (B) MKI67 mRNA expression is down-regulated by SKSI-0412 in IL-6-induced keratinocyte for 3 hrs. *P < 0.05, **P< 0.01 compared with non-treated control; ## P<0.01, #### P<0.0001 compared with IL-6 treatment sample, ANOVA one-way test. (C) Proliferation marker protein Ki-67 expression was analyzed using immunofluorescence staining of Ki-67 (green) and DAPI (blue). *Scale bar* =100 μm. (D) Cell proliferation was detected at 24 and 48 hrs. **P< 0.01, ****P< 0.0001 compared with non-treated control; ### P<0.001, #### P<0.0001 compared with IL-6 treatment sample, ANOVA one-way test.
